# Using Risk Scores to Estimate Lower Extremity Fragility Fracture Risk among Individuals with Chronic Spinal Cord Injury: A Preliminary Model

**DOI:** 10.46292/sci23-00063S

**Published:** 2023-11-17

**Authors:** B. Catharine Craven, Lora M. Giangregorio, Isabelle Côté, Lindsie Blencowe, Masae Miyatani, Mohammad Alavinia

**Affiliations:** 1 KITE Research Institute, University Health Network. Toronto, ON, Canada; 2 Department of Medicine, Temerty Faculty of Medicine, University of Toronto, Toronto, ON, Canada; 3 Department of Kinesiology and Health Science, University of Waterloo, Waterloo, ON, Canada; 4 Rehabilitation Sciences Institute, Temerty Faculty of Medicine, University of Toronto, Toronto, ON, Canada; 5 Institute of Health Policy Management and Evaluation, University of Toronto, Toronto, ON, Canada; 6 CIUSSSCN - Institut de Réadaptation en Déficience Physique de Québec, Canada

**Keywords:** fracture, osteoporosis, risk score, spinal cord injuries

## Abstract

**Objectives:**

To develop SCI-FX, a risk score to estimate 5-year lower extremity fragility fracture risk among patients living with chronic spinal cord injury (cSCI).

**Methods:**

Adults with traumatic cSCI (*n* = 90) participated in a 2-year prospective longitudinal cohort study describing bone mineral density (BMD) change and fracture incidence conducted at the Lyndhurst Centre (University Health Network), University of Waterloo, and Physical Disability Rehabilitation Institute of Québec City. Prior publication and clinical intuition were used to identify fragility fracture risk factors including prior fragility fracture, years post-injury, motor complete injury (AIS A/B), benzodiazepine use, opioid use, and parental osteoporosis. We conducted bivariate analyses to identify variables associated with fracture. Multiple logistic regressions were performed using fragility fracture incidence as the dependent variable and all variables from the univariate analyses with a highly liberal *p* value at 0.2. Using the odds ratios (ORs) from the multiple logistic regression model, a point system for fragility fracture risk score was developed, and the odds of fracture for each point was estimated.

**Results:**

All initial variables, with the exception of benzodiazepine exposure, were included in the final model.

**Conclusion:**

We identified a simple preliminary model for clinicians to estimate 5-year fracture risk among patients with cSCI based on their total score.

## Introduction

Within 18 months of spinal cord injury (SCI) onset, individuals with motor complete SCI experience an extensive (30%-50%) loss of hip, distal femur, and proximal tibia bone mineral density (BMD), predisposing them to a lifetime increased risk of lower extremity fragility fracture. The lifetime incidence of fracture ranges from 25% to 50%.^1^-^4^ Fracture rates vary between 2.2 and 3.2 fractures per 100 patient-years.^1^,^3^,^5^-^7^ The median time to first fracture is 8.5 years after injury onset.^4^ Fractures of the proximal tibia, distal femur, and hip are the most common, in descending order of frequency. Fragility fractures after SCI often occur at home after a fall from standing or seated height onto a flexed knee or from lower limb rotational stress during activities of daily living such as a car transfer.^8^-^13^ Females over age 50 are at highest risk of fracture compared to males with SCI at any age (hazard ratio [HR] = 1.54; 95% CI: 1.12-2.11).^14^

Fractures after SCI often do not heal well. Many individuals experience poor outcomes, including wound, pin site or joint infection, delayed union or nonunion, segmental shortening, and amputation. Further, fractures increase morbidity due to concurrent health conditions, including pressure injury, venous thromboembolism, urinary tract infections, respiratory infections, delirium,^1^,^2^ autonomic dysreflexia, spasticity,^15^ and depression.^14^,^16^ Carbone et al. have reported an increase in 5-year mortality among male Veterans (*n* = 12,389) living with chronic traumatic SCI and lower extremity fractures in the United States.^17^ This risk of mortality is greatest in males over age 50 (HR = 3.42; 95% CI: 2.8-4.25).^3^,^17^

Newly developed SCI-specific position statements regarding BMD testing after SCI from the International Society for Clinical Densitometry (ISCD)^18^ and the Paralyzed Veterans of America's Bone Health and Osteoporosis Management Clinical Practice Guidelines^19^ stipulate that “clinicians should consider an individual's fracture risk” before selecting therapy to reduce fracture risk^20^,^21^ and should consider an individual's safety for participation in rehabilitation therapy or recreational activity.^18^

When fracture risk is high, (1) pharmacotherapy (bisphosphonates or denosumab) with dietary calcium and vitamin D supplements are recommended to augment hip or knee region (distal femur and proximal tibia) BMD,^22^-^24^ and (2) prescreening of fracture risk, informed consent, and caution are recommended prior to implementing rehabilitation interventions such as functional electrical stimulation, neuromuscular electrical stimulation, body weight support treadmill training, or exoskeleton therapy.

The conundrum for clinicians is that available tools used to estimate fracture risk in the general population to guide therapeutic decision-making are not valid for individuals with SCI and do not capture SCI-specific risk factors for fracture.^25^ Thus, clinicians are left to assign fracture risk based on their clinical judgment, or they use tools to calculate fracture risk that were designed to identify osteoporosis in the general population of postmenopausal women or aging men over age 40, specifically the Fracture Risk Assessment Tool (frax^®^).^26^

FRAX is the most widely used tool internationally for estimating 10-year fracture risk in the general population. FRAX has been validated for use in Canada^27^ as the Canadian Association of Radiologists and Osteoporosis Canada (CAROC)^28^ tool. FRAX may be used with and without BMD to calculate fracture risk, whereas CAROC requires femoral neck BMD T-scores to estimate fracture risk. Neither tool considers risk factors unique to individuals with SCI nor the age of SCI onset or duration of injury. FRAX assumes all individuals are at least 40 years of age, whereas CAROC assumes all individuals are at least 50 years of age and less than 85 years of age. In the general population, fracture risk increases with age. In SCI, there is a bimodal age distribution: one distribution includes young men and premenopausal women under age 50 who experience motor vehicle accidents or trauma (i.e., gunshots, sports injuries), and a second distribution includes older adults with existing osteoporosis who experienced a nontraumatic etiology of their SCI.^29^

A prior study from our lab reveals poor agreement between these existing fracture risk assessment tools when used in individuals with SCI.^25^ Fracture risk stratification varied with age, sex, and tool used, demonstrating validity concerns regarding application of CAROC and FRAX in the SCI population.^25^ Specifically, we compared 10-year fracture risk using CAROC and FRAX and compared knee-region BMD and distal tibia volumetric BMD to SCI-specific fracture thresholds. Agreements between CAROC and FRAX risk stratifications and between fracture threshold risk stratification were assessed using prevalence and bias-adjusted Kappa statistics (PABAK). There was moderate agreement between the CAROC and FRAX tools in postmenopausal women (PABAK = 0.56; 95% CI: 0.27-0.84) and men aged ≥50 years (PABAK = 0.51; 95% CI: 0.34-0.67) but poor agreement for young men and premenopausal women (PABAK ≤ 0), who are a large proportion of people living with SCI. This indicates that current fracture risk tools do not identify individuals with SCI who have low hip and knee region BMD as “at high risk for fracture,” precluding physicians from referring them for BMD testing or initiating drug therapy.^25^

Thus, there is an urgent and compelling need for a SCI-specific fracture risk prediction tool based on clinical risk factors for fracture. Ideally, this tool would allow for estimation of 5-year fracture risk without BMD testing, as access to bone density testing at the hip and knee regions is not universal at this time. The aim of this study is to develop a risk score to estimate 5-year fragility fracture risk among individuals living with chronic SCI (cSCI).

## Method

### Study design

Community-dwelling adults with cSCI of traumatic etiology were recruited as part of a multicentre 2-year prospective observational cohort study.^30^ The study assessments consisted of medical history, including injury etiology and impairment descriptors, concurrent medical conditions, medication review, and fracture ascertainment among other assessments. These assessments occurred at baseline and 6, 12, 18 and 24 months. Occurrence of a new fragility fracture during the follow-up period was examined relative to the baseline medical, demographic, and impairment variables to develop a risk score to estimate fracture risk among adults living with cSCI.

### Participants

Participants with SCI were enrolled at the clinics at tertiary SCI rehabilitation hospitals in the Canadian provinces of Ontario (*n* = 70) and Québec (*n* = 20). Study recruitment began in March 2009, and data were collected between April 2009 and June 2014. Adult participants (≥ 18 years old) with a cSCI (C2-T12, American Spinal Injury Association Impairment Scale [AIS] A-D) of traumatic etiology for at least 2 years prior to enrollment were recruited. All participants provided written informed consent. Participants were excluded if they had current or prior conditions other than paralysis known to adversely influence bone metabolism, a weight greater than 123 kg (densitometer limit), or were pregnant or planning to become pregnant. The relevant institutional research ethics boards in Ontario and Québec approved the study protocol.

### Lower extremity fragility fracture

The processes for using medical record data to verify fractures are based on those used within the Canadian Multicentre Osteoporosis Study (CaMOS Study)^31^ and include completion of a fracture ascertainment questionnaire every 6 months during study visits (baseline and 12 months and 24 months) and during follow-up phone calls (6 months and 18 months). Participants reported the cause and location of any interim fractures. Written informed consent was obtained from participants for obtaining diagnostic imaging reports and consult notes to verify fracture occurrence, location, and severity. Fragility fractures were those that occurred due to minimal or no trauma. Fractures caused by high-energy trauma or fractures that occurred prior to injury, or at the time of the injury, were removed from the dataset and excluded from the analysis. For the purpose of this analysis, only lower extremity fractures were included.

### Sociodemographic and clinical characteristics

Demographic, current and past health status, medication use, and impairment data were obtained by participant interview, examination, or chart abstraction at baseline. Subsequent information regarding health status and medications was obtained at 12- and 24-month follow-up visits and at 6- and 18-month phone calls. Physiatrists determined neurological level of injury, AIS category, and motor and sensory scores using the International Standards for Neurologic Classification of Spinal Cord Injury at the baseline.^32^ Time post injury (years) was calculated as the date of injury minus the date of baseline medical history assessment.

### Statistical analysis

Continuous variables are reported as mean (*SD*), and categorical variables are presented as number and frequency. Important nonmodifiable and modifiable risk factors for fragility fracture among SCI individuals were identified based on clinical experience and prior research, which included the variables shown in Table 1.^33^-^37^ To make the models as easy as possible to use in clinical practice, we restricted our attention to risk factors that are readily available or supported by evidence in clinical practice without BMD testing. Potential risk factors were the age at injury, years post injury, history of fragility fracture, parental osteoporosis, opioid use, benzodiazepine use, and current smoking status. Univariate logistic regressions were performed using fragility fracture as the dependent variable and each of these aforementioned six risk factors to determine the odds of fracture. Variables with the odds ratio (OR) at a liberal *p* value of 0.2 were entered into the reverse multiple regression model. The threshold of 0.2 allowed for a higher tolerance for type I errors to capture a broader set of potential predictors. We considered the reference category to be 0 risk for each risk factor, and then calculated how far each category was from the reference category in regression units. The maximum score added across risk factors was 21, with a probability of 5-year lower extremity fracture reported for each score.

**Table 1.** Risk factors for lower extremity fragility fracture after chronic spinal cord injury

Risk factors	
1.	Prior lower extremity fracture^33^
2.	Alcohol intake >5 servings per day^36^
3.	Current smoker*
4.	Paraplegia^33^,^37^
5.	Duration of SCI ≥10 years^35^
6.	Motor complete injury (AIS A-B)^34^,^37^
7.	Osteoporosis in a first degree relative*
8.	Hip fracture in the last year
9.	Routine use of benzodiazepines
10.	Routine use of opioid analgesia (≥28 mg morphine for a 3-month period)^33^-^35^

*Note:* Risk factors 3, 4, 5, 6, and 9 were included in the multivariable models. Risk factor 1 was used as a covariate. Risk factor 6 was converted to osteoporosis in a first-degree relative. The risk factors with an asterisk (*) are derived from clinical intuition. AIS = American Spinal Injury Association Impairment Scale.

To define risk strata, we took the midpoint of each category as the reference value and then selected a referent risk factor profile by choosing a base category for each risk factor and assigning it a score of 0. Risk factors were assigned positive points; a higher point total will convey greater fracture risk (see Table 4). We assigned 0 points to the following categories: no prior fragility fracture, 0-9 years post injury, AIS CD, no history of parental osteoporosis, and no opioid use. We then calculated how far each category was from the base in regression units, [βi (W_ij_ − W_iREF_)] for each risk factor (see Table 4). Subsequently, we defined the constant for the points system—the number of regression units that correspond to one point in the model. For this purpose, we considered *B* as the increase in risk associated with a 5-year increase in time post injury, where *B* = 5 * 0.1059 = 0.5295. Points associated with each risk factor category were calculated by Points_ij_ = β_i_(W_ij_ − W_iREF_)/*B* and rounded to the nearest integer. We determined the risks associated with the total points for each patient. The total points range from 0 to 21, and the probability of the risk is computed for each point to determine an individual's risk (the probability of developing a fracture) using the formula:


$$\rho =\frac{1}{1+\text{exp}(-\sum bixi)}$$

where ∑βixi = intercept + β_1_x_1_+β_2_x_2_+β_3_x_3_+β_4_x_4_+β (point total).

We approximated ∑βixi as:

∑βixi = −9.8179 + 0.1417 (4.5) + 0 + 0 + 0+ 0.7085 (point total).

The coefficients represent the change in the log-odds of fragility fracture for a one-unit change in the corresponding predictor variable while keeping the other variables constant. The risk score denotes the estimated risk of a fragility fracture in the context of medical diagnosis or risk assessment. The higher the risk score, the higher the estimated probability of fragility fracture.

As cSCI is a relatively rare disease, the OR can be used as an estimate of relative risk.^38^

The final model quantifies the impact of multiple risk factors on the odds of 5-year lower extremity fracture among patients with cSCI based on a combination of clinically available risk factors and the odds of fracture. These variables included prior fragility fracture, time post injury, motor complete injury (AIS AB vs. CD), parenteral osteoporosis, and opioid use. Age and prior fracture were used as covariates in several multivariable models; we chose to use duration of injury as opposed to age as the covariate in the final model.

As cSCI is a relatively rare disease, OR and not relative risk (RR) for incident fractures was reported. The final multivariable model essentially uses the ORs from the multiple logistic regression model to create a point system for calculating^39^,^40^ the probability of fracture for each point in the risk assessment score.

Classification and regression tree (CART) analysis was used to develop a preliminary model that can classify subjects into various risk categories.^41^,^42^ In this analysis, the variables that remained statistically or clinically significant were entered into a CART analysis. All statistical analyses were performed on SAS 9.4 (SAS Institute, Cary, North Carolina).

## Results

During recruitment, 453 individuals were approached for study participation; of these 453 individuals, 288 were unreachable, deceased, or declined participation. Following the screening, 90 consenting adults (64 men and 26 women), 70 from Ontario and 20 from Québec, were eligible for participation. Table 2 shows the demographic and impairment characteristics of the study cohort. The majority of participants had an AIS A score (60.0%), with a mean time post injury of 15.23 years (*SD* = 9.58). Thirteen participants had 36 fragility fractures during the follow-up period, 10 had more than one fracture, and three participants reported multiple fractures (five or more). The fracture locations among the three participants were hip, knee, femur, tibia, ankle, and ribs.

**Table 2.** Baseline characteristics of the 90 participants with chronic spinal cord injury

Demographic characteristics	Mean (*SD*) or *n* (%)
Age, years	47.8 (12.1)
Age at SCI, years	32.58 (14.13)
Years post injury, years	15.23 (9.58)
Female	26 (28.89%)
AIS-Paraplegia	
A	26 (29%)
B	4 (4%)
C	7 (8%)
D	6 (7%)
AIS-Tetraplegia	
A	28 (31%)
B	2 (2%)
C	7 (8%)
D	10 (11%)
Prior fragility fracture	22 (24.44%)
Parental osteoporosis	17 (19.54%)
Opioid use	21 (25.00%)
Benzodiazepine	21 (23.33%)
Fractures in follow-up period	36

*Note:* This cohort has a larger number of participants and participants with fracture than described in our initial manuscripts. AIS = American Spinal Injury Association Impairment Scale.

Table 3 shows the odds ratios derived from the univariate and multivariable logistic regression among the cSCI cohort. Table 4 displays the calculated point value for each risk factor for lower extremity fracture among participants with cSCI. The highest score belongs to prior fragility fracture (score of 7), and the lowest belongs to motor complete injury (score of 1). Table 5 displays the SCI-FX fracture risk assessment tool. Table 6 displays the point system for calculating the total score and the 5-year estimated fracture risk based on the individual's total score. For example, if an individual with motor complete SCI of 10 years duration, taking opioids, has a parent with a prior hip fracture, their 5-year fracture risk is estimated to be 20% based on a preliminary SCI-FX score of 11.

**Table 3.** Univariate and multivariable logistic regression for prediction of fragility fracture among 50 individuals with chronic spinal cord injury

Risk factors	Univariate logistic regression		Multivariable logistic regression	
	OR	*p* value	OR	*p* value
Prior fragility fracture	80.397	<.0001	32.290	.0007
Years post injury	1.099	.005	1.112	.047
AIS A & B	7.249	.06	2.148	.5
Benzodiazepines^a^	3.543	.04	—	—
Opioid	2.5	.159	4.236	.13
Parental	3.229	.072	7.167	.06
osteoporosis				
Intercept	—	—	7.0631	

*Note:* AIS = American Spinal Injury Association Impairment Scale; OR = odds ratio.

a Benzodiazepines were excluded from the final model in the backward logistic regression method.

**Table 4.** Calculating the point value for each lower extremity fracture risk factor

Risk factor	Categories	Reference value	β_i_	Βi (Wij-WiREF)	Scoreβi(W_ij_ − W_iREF_)/B.
Prior fragility fracture	No	0	3.4748	0	0
	Yes	1		3.4748	7
Years post injury^a^	0-9	4.5	0.1059	0	0
	10-19	14.5		1.059	2
	20-29	24.5		2.118	4
	≥30	35.5		3.2829	6
AIS score	CD	0	0.7645	0	0
	AB	1		0.7645	1
Parenteral osteoporosis	No	0	1.9694	0	0
	Yes	1		1.9694	4
Opioid use	No	0	1.4437	0	0
	Yes	1		1.4437	3

*Note:* AIS = American Spinal Injury Association Impairment Scale.

a The years post injury range in the sample is 2 to 41 years post injury.

**Table 5.** Scores associated with each SCI-FX risk factor category

Risk factor	Category	Score
Prior fracture	No	0
	Yes	7
Years post injury	0-9	0
	10-19	2
	20-29	4
	≥30	6
AIS (CD vs. AB)	CD=0	0
	AB=1	2
Parental osteoporosis	No	0
	Yes	4
Opioid use	No	0
	Yes	3
Total score		/22

*Note:* AIS = American Spinal Injury Association Impairment Scale.

**Table 6.** Five-year risk estimation for lower extremity fragility fracture based on an individual's total score from Table 5

Point total	Estimate of risk	Point total	Estimate of 5-year fracture risk
0	0.0014	10	0.1100
1	0.0023	12	0.4421
2	0.0040	13	0.5737
3	0.0067	14	0.6956
4	0.0113	15	0.7951
5	0.019	16	0.8682
6	0.032	17	0.9180
7	0.053	18	0.9500
8	0.087	19	0.9699
9	0.1393	20	0.9821
10	0.2156	21	0.9894
11	0.3182		

**Figure 1.**
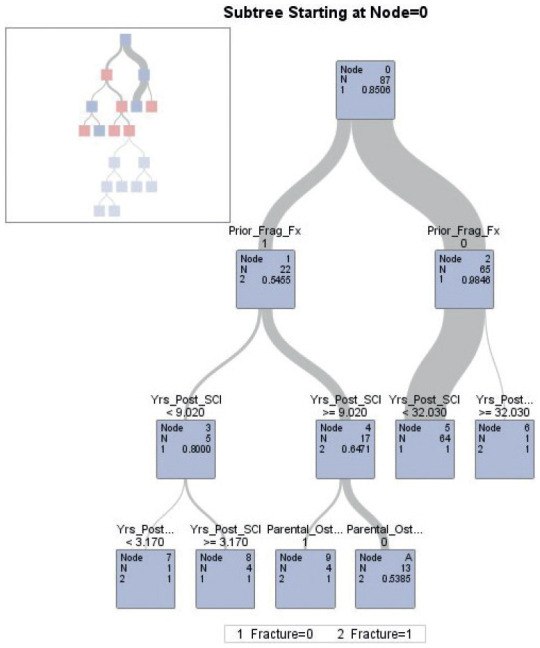
Risk score calculations for the significant nodes of the classification and regression tree (CART) analysis. Prior_Frag_FX = prior fragility fracture; Yrs_Post_SCI = years post injury.

**Figure 1** shows the CART decision tree to predict fragility fracture for this sample. The area under the curve (AUC) was 0.998. The model has a sensitivity of 1, which means that all patients at risk of fragility fracture could be diagnosed. It also could identify a high proportion of negative cases (specificity = 0.92). The low entropy value of 0.05 suggests that the decision tree makes very pure splits, resulting in well-separated classes at the leaf nodes. Based on the sensitivity and specificity and the calculated precision, the F1 score for the model is approximately 0.957, which implies that the model's performance is well-balanced between precision and recall, considering both true positive and false positive rates.

CART analysis defined two risk factors categorized into four nodes, prior fragility fracture and years post injury. In this model, the probability of imminent fracture in 2 years among patients with a history of fracture and more than 9 years post injury was 65% (see **Figure 1**).

## Discussion

Although risk factors for lower fragility fracture among individuals with cSCI are well known, evidence-based decision-support tools for stratification of individuals with SCI regarding their risk of fracture are not currently available. We defined a simple model using clinical risk factors for identifying individuals with cSCI at risk for lower extremity fragility fracture and can quantify their 5-year fracture risk using their total score. We intentionally restricted our attention to risk factors that are generally accepted and readily available in clinical practice. The most potent risk factors for fractures in descending order of odds ratios include prior fracture, parental osteoporosis, opioid use, motor complete injury, and years post injury. These risk factors are consistent with prior publications, although only opioid exposure is a readily modifiable risk factor.

Using the risk score model, a clinician can estimate a patient's 5-year risk of lower extremity fracture based on their response to five simple questions and their associated point values (Table 5):

1. Have you had a prior fragility fracture?
2. How many years since the onset of your SCI?
3. Does your mother or father have osteoporosis?
4. Do you have a motor complete versus an incomplete injury? Do you know your AIS category?
5. Do you regularly use opioids?

The individual's fracture risk score is then estimated by adding the risk scores associated with the individual's risk factor profile and then looking up the odds of fracture associated with 5-year fracture risk (Table 5). This estimate of 5-year lower extremity fragility fracture risk among adults with cSCI will help physicians to identify patients at risk for fracture who may benefit from nutraceutical, drug, or rehabilitation interventions alone or in combination to augment bone mass and ameliorate fracture risk.

Predicting the risk of a health problem is an important issue in clinical practice. The accurate estimation of risk allows for effective clinical decision-making and classification of the patients based on their individual future risk of the problem (fracture). Our model is a preliminary one and is not ready for clinical implementation at this time as the small sample size could result in an overestimation of the risk. We have secured peer-reviewed funding to validate the model using Canadian civilian and US Veterans Affairs data over the next 2 years.

We present the CART analysis to help clinicians appreciate the value of dichotomizing data for decision-making purposes. In the future, we plan to elaborate the CART analysis with a much larger data set. A valid risk classification tool has the potential to promote health system efficiency and increase the effectiveness of the care provider's and care recipient's decision-making processes. Using the preliminary model, the prognosis for a health problem (lower extremity fracture) can be reported based on the total score assigned to each patient.

Some specific limitations of this preliminary SCI-FX model for calculating 5-year fracture risk should be considered. First, due to the small sample size, we were not able to validate the risk score using a separate patient cohort; this is a planned area for future research. The sample size (*n* = 90) limited the number of predefined risk factors that could be examined in the multiple variable models. Second, this is only a preliminary model with a limited sample size and is insufficient to be implicated as a clinical tool. Nonetheless, it can guide future development of the tool, and it can be validated internally (test data set) and externally. Third, although the data were from a prospective cohort of individuals with cSCI, the exact time of the fragility fracture during the follow-up period was not always certain. Therefore, we could not calculate the risk of fragility fracture using a survival analysis. Finally, only two variables were included in the CART analysis. CART analysis is most suitable with a sample size that includes 3 to 20 times the number of variables and absolute ranges from 100 to over 1000.^43^

Due to the altered bone metabolism and high complication rates in patients with SCI, the management of fractures needs special consideration; even with appropriate treatment, nonunion is a common complication in these patients.^1^,^36^,^44^ Therefore, identifying SCI individuals who are at high risk for the fracture and who would benefit from interventions to maintain bone mass and reduce fracture risk is a high priority.

In conclusion, this current preliminary model provides clinicians with a simple risk score calculator to quickly determine a patient's 5-year risk of developing fragility fracture among patients with cSCI. This, in turn, should result in timely physician assessment and early intervention to modify bone architecture prior to fracture incidence. Further model elaboration is required prior to routine clinical implementation of this preliminary fracture risk prediction model.
